# Impact of air pollutants on pediatric admissions for *Mycoplasma* pneumonia: a cross-sectional study in Shanghai, China

**DOI:** 10.1186/s12889-020-8423-4

**Published:** 2020-04-06

**Authors:** Ning Chen, Jianwei Shi, Jiaoling Huang, Wenya Yu, Rui Liu, Li Gu, Rong Yang, Zhaohu Yu, Qian Liu, Yan Yang, Sainan Cui, Zhaoxin Wang

**Affiliations:** 1 Department of Pediatrics, Shanghai Tenth People’s Hospital, Tongji University School of Medicine, Shanghai, 200072 China; 2 School of Medicine, Tongji University, Shanghai, 200092 China; 3 School of Public Health, Shanghai Jiaotong University School of Medicine, 227 South Chongqing Rd, Shanghai, 200025 China; 4 Yangpu Hospital, Tongji University School of Medicine, Shanghai, 200090 China; 5 Navy 971 Hospital, Qingdao, 266071 China; 6 School of Economics & Management, Tongji University, Shanghai, 200092 China; 7 General Practice Center, Nanhai Hospital, Southern Medical University, Foshan, 528244 China

**Keywords:** *Mycoplasma* pneumonia, Air pollutants, Nitrogen dioxide, Fine particles, Children health

## Abstract

**Background:**

Children are especially vulnerable to pneumonia and the effects of air pollution. However, little is known about the impacts of air pollutants on pediatric admissions for *Mycoplasma* pneumonia. This study was conducted to investigate the impacts of air pollutants on pediatric hospital admissions for *Mycoplasma* pneumonia in Shanghai, China.

**Methods:**

A cross-sectional design was applied to explore the association between pediatric hospital admissions and levels of air pollutants (fine particulate matter, particulate matter, ozone, sulfur dioxide, nitrogen dioxide, and carbon monoxide). Data on hospital admissions for pneumonia and levels of ambient air pollutants were obtained for the period of 2015 to 2018. Associations between pediatric admissions for *Mycoplasma* pneumonia and ambient air pollutants were calculated using logistic regression and described by the odds ratio and relevant 95% confidence interval. The hysteresis effects of air pollutants from the day of hospital admission to the previous 7 days were evaluated in single-pollutant models and multi-pollutant models with adjustments for weather variables and seasonality. Lag 0 was defined as the day of hospital admission, lag 1 was defined as the day before hospital admission, and so forth.

**Results:**

In the single-pollutant models (without adjustment for other pollutants), pediatric hospital admissions for pneumonia were positively associated with elevated concentrations of nitrogen dioxide and fine particulate matter. A 0.5% increase in daily admissions per 10-μg/m^3^ increase in the nitrogen dioxide level occurred at lag 1 and lag 2, and a 0.3% increase in daily admissions per 10-μg/m^3^ increase in fine particulate matter occurred at lag 1. In the multi-pollutant models, nitrogen dioxide and fine particulate matter remained significant after inclusion of particulate matter, ozone, sulfur dioxide, and carbon monoxide.

**Conclusions:**

This study illustrated that higher levels of nitrogen dioxide and fine particulate matter increase the risk of pediatric hospitalization for *Mycoplasma* pneumonia in Shanghai, China. These findings imply that the high incidence of *Mycoplasma* pneumonia in children in Asia might be attributed to the high concentration of specific air pollutants in Asia.

## Background

Pneumonia is the single largest infectious cause of death in children worldwide, accounting for 16% of all deaths of children aged < 5 years and accounting for the deaths of 920,136 children in 2015 [1]. *Mycoplasma pneumoniae* is a leading causative pathogen of respiratory infections in children, accounting for as many as 30% of cases of pediatric community-acquired pneumonia [2–5]. In 2012, Miyashita et al. demonstrated that *M. pneumoniae* was the most prevalent pathogen in children (23%) and adolescents (29%) with community-acquired pneumonia, followed by *Haemophilus influenzae* (children, 15%; adolescents, 10%) and *Streptococcus pneumoniae* (children, 8%; adolescents, 14%) [6]. In 2018, Liu et al. reported that *M. pneumoniae* was detected at the highest frequency in pediatric patients hospitalized for lower respiratory tract infections (15.7%), followed by respiratory syncytial virus (13.9%) [7]. The underlying mechanisms of *M. pneumoniae* pneumonia are currently being investigated. Nitrogen dioxide (NO_2_) has been conjectured to be associated with bacterial pneumonia. For instance, according to toxicological studies released by the United States Environmental Protection Agency, NO_2_ destroys epithelial cells and decreases mucociliary clearance, thereby reducing the amount of bronchial macrophages, natural killer cells, and macrophages as well as the CD4-to-CD8 ratio and ultimately enhancing susceptibility to bacterial pathogens [8]. Additionally, a 2017 meta-analysis regarding the association between ambient air pollution and pediatric pneumonia showed that the pollutant-specific excess risk percentage per 10-ppb increase in gaseous pollutants was 1.4% [95% confidence interval (CI), 0.4–2.4%] for NO_2_ [9]. Apart from NO_2_, fine particulate matter [particles of < 2.5 μm in aerodynamic diameter (PM_2.5_)] has also been speculated to be associated with pneumonia. A case-crossover study conducted in Shijiazhuang, China, showed a positive correlation between hospitalization for pneumonia and higher PM_2.5_ levels in both single-pollutant and multi-pollutant models [10]. In 2016, Patto et al. reported that a 10-μg/m^3^ decrease in the PM_2.5_ concentration was associated with 256 fewer pediatric hospital admissions for pneumonia [11]. In 2019, Croft et al. showed that increases in the interquartile range of PM_2.5_ during the previous week were correlated with increases in the excess rate of hospitalizations for culture-negative pneumonia (2.5%; 95% CI, 1.7–3.2) as well as the excess rate of hospitalizations for bacterial pneumonia (2.3%; 95% CI, 0.3–4.3) [12]. However, the mechanisms underlying how these air pollutants cause pediatric pneumonia remain unclear.

The health of the Chinese population is threatened by the high level of air pollutants caused by industrialization and urbanization [13]. According to data published by the Chinese Ministry of Environmental Protection, most cities in China currently fail to meet the following standard daily air pollutant concentrations: PM_2.5_ of 35 μg/m^3^, particulate matter [particles of < 10 μm in aerodynamic diameter (PM_10_)] of 50 μg/m^3^, ozone (O_3_) of 100 μg/m^3^ (8-h average), sulfur dioxide (SO_2_) of 50 μg/m^3^, NO_2_ of 80 μg/m^3^, and carbon monoxide (CO) of 4 mg/m^3^ [14]. Additionally, some of the annual air pollution levels (2015–2018) have exceeded the recommended standards proposed by the World Health Organization, with a PM_2.5_ of 44.75 μg/m^3^ (higher than the recommended level of 10 μg/m^3^), PM_10_ of 78.75 μg/m^3^ (far beyond the recommendation of 20 μg/m^3^), and O_3_ level of 143 μg/m^3^ (higher than the recommendation of 100 μg/m^3^). The annual level of SO_2_ in China from 2015 to 2018 was 19.75 μg/m^3^ and that of CO was 1.8 mg/m^3^, while the level of NO_2_ was 30 μg/m^3^ (slightly lower than 40 μg/m^3^).

As a rapidly urbanizing city in the Yangtze River Delta in China, Shanghai has been experiencing problems caused by environmental pollution with concentrations of main air pollutants far exceeding the national standards for years. The major sources of air pollution are automobile exhaust emissions and industrial waste gases. According to the China Mobile Source Environmental Management Annual Report (2019), mobile sources have become the major sources of air pollution, especially PM_2.5_, in large and medium-sized cities such as Shanghai, Beijing, and Shenzhen. Children are more vulnerable to the respiratory health effects of ambient air pollution because of their frequent exposure to outdoor air, high respiratory rate, narrow airways, and developing lungs and immune systems [15]. Although many studies have demonstrated the potential adverse health effects attributed to air pollutants, including cardiovascular mortality [16], asthma exacerbation [17], and restricted activity [18], few empirical studies have proven the relationship between levels of air pollutants and *Mycoplasma* pneumonia in the pediatric population.

Because of the lack of empirical studies regarding the influence of air pollutants on *Mycoplasma* pneumonia among children, we performed the present cross-sectional study to examine the impacts of air pollutants on hospital admissions for *Mycoplasma* pneumonia and elucidate the hysteresis effects of air pollutants among children aged 1 to 14 years in Shanghai, a large modern city in southeast China.

## Methods

### Study design and setting

In this descriptive cross-sectional study, we investigated variations in pediatric *Mycoplasma* pneumonia in relation to ambient air pollutant levels in Shanghai throughout a 4-year period (2015–2018). Shanghai is one of the largest metropolitan cities in the world. Its gross domestic product ranks first in China, and its population in 2017 was approximately 24.18 million. The city enjoys a subtropical humid monsoon climate, and the mean annual temperature was 18.34 °C ± 8.71 °C from 2015 to 2018.

### Data collection

We extracted data on all hospital admissions of inpatients aged 1 to 14 years who were admitted with a primary diagnosis of pneumonia (International Classification of Diseases, 11th revision code: CA40.04) from January 2015 through December 2018. Patient data were obtained from the Pediatric Department of Shanghai Tenth People’s Hospital, one of the largest tertiary general hospitals in Jing’an District of Shanghai. This hospital receives a large proportion of the pediatric inpatients in this area. Data from 2569 eligible patients were obtained from pediatric admissions for pneumonia in this hospital. Among them, 693 patients were diagnosed with *Mycoplasma* pneumonia. *Mycoplasma* pneumonia antibody was detected by colloidal gold assay. Age, sex, and date of admission were extracted from the medical records.

Data on the following air pollutants in Shanghai were obtained from the website of the Shanghai Environmental Monitoring Center: PM_2.5_, PM_10_, O_3_, SO_2_, NO_2_, and CO. These data were recorded every 1, 8, or 24 h. The 24-h average levels of all of these pollutants except O_3_ (maximum 8-h average level) were applied in our analysis. Nine air quality monitoring stations were employed by the Shanghai Municipal Bureau of Ecology and Environment. The sampling points were located in Putuo District, Yangpu District, Huangpu District, Hongkou District, Jing’an District, Xuhui District, and Pudong New area. The exposure levels were calculated as the mean monitoring data of the nine stations and were disclosed by the Shanghai Environmental Monitoring Center every 1 h and every 24 h separately.

Daily temperature data in Shanghai were collected from the Shanghai Meteorological Service. Monthly relative humidity data were compiled from the China Statistical Yearbook by the National Bureau of Statistics.

### Study variables

The results of *M. pneumoniae* immunoglobulin M detection were considered as the dependent variable (*M. pneumoniae*-positive = 1, *M. pneumoniae*-negative = 0). The control variables were age, sex, and season of admission.

We considered several independent variables, including pollutants and climatic variables, for the logistic regression model. The concentrations of the main air pollutants were calculated using 24-h averages. We calculated the monthly averages for further analysis. The unit of measurement for PM_2.5_, PM_10_, O_3_, SO_2_, and NO_2_ was μg/m^3^, and the unit of measurement for CO was mg/m^3^. Time was considered to be a potential confounder because of its mixed relationship with air pollutants. Thus, the models included indicator variables for season (spring, summer, autumn, and winter) and lag days of a week (7 days). The daily mean temperature and monthly relative humidity were also included in the analysis.

### Statistical analysis

A time-stratified approach was utilized to select control days. For the exposure metrics, the air pollutant levels of every hospital admission day were compared with those of the whole week before the date of admission. The impacts on pediatric hospital admission for pneumonia were assessed with lags from day 0 to the previous 7 days. Lag 0 was defined as the day of hospital admission, lag 1 was defined as the day before hospital admission, and so forth.

Descriptive statistical analyses of all variables were performed to characterize the features of the patients, air pollutants, and meteorological data separately. Associations between pediatric hospital admissions for pneumonia and ambient air pollutant variables of interest were calculated using logistic regression, described by the odds ratio (OR) and relevant 95% CI. To evaluate the impacts of atmospheric pollutants on pediatric hospital admissions for *Mycoplasma* pneumonia, single-pollutant and multi-pollutant models were used to calculate the associations between them. Single-pollutant models were initially employed to evaluate the effects of each air pollutant. Then multi-pollutant models including all the six pollutants were also performed to present the condition of mixed states of these pollutants. Concentrations of air pollutants were included in the models as continuous variables. The model controlled for meteorological variables that might function as the prime potential confounding factors (daily mean temperature and relative humidity). We then calculated the elevated risk of pediatric admission for *Mycoplasma* pneumonia with the corresponding 10-μg/m^3^ or 1**-**mg/m^3^ increase the air pollutant concentration. Stratified analyses of pollutant exposure on the basis of age, sex (male or female), and season (spring: March–May; summer: June–August; autumn: September–November; winter: December–February) were applied to estimate the effect modification.

All statistical analyses were performed using IBM SPSS Statistics version 21 (IBM Corp., Armonk, NY, USA). The criterion for significance was set at *p* < 0.05.

## Results

### Descriptive statistical analysis

A total of 2569 admissions to the Pediatric Department of Shanghai Tenth People’s Hospital for *M. pneumoniae* detection occurred from 2015 through 2018. The characteristics of the pediatric hospital admissions categorized by sex, age, and season are presented in Table 1. Among the 2569 admissions, 1281 (49.86%) patients were male and 1288 (50.14%) were female. The children’s ages ranged from 1 to 14 years (median, 4 years). In total, 693 patients were diagnosed with *Mycoplasma* pneumonia (Table 1). No significant difference was found in age or sex between children who were and were not diagnosed with pneumonia (*p* > 0.05 for both). However, significant differences were found in the season of pediatric hospital admissions between the two groups (*p* < 0.05 for all seasons).

**Table 1:** Distribution of pediatric hospital admissions for pneumonia based on patient characteristics and season (*n* = 2569)

Characteristics	MP-Positive		MP-Negative		Total Admission	
	Number	Percentage(%)	Number	Percentage(%)	Number	Percentage(%)
**Gender**						
Male	331	47.76	950	50.64	1281	49.86
Female	362	52.24	926	49.36	1288	50.14
**Age (year)**						
1–4	249	35.93	1065	56.77	1314	51.15
5–14	444	64.07	811	43.23	1255	48.85
**Season**						
Spring	156	22.51	489	26.07	645	25.11
Summer	111	16.02	241	12.85	352	13.70
Autumn	210	30.30	502	26.76	712	27.72
Winter	216	31.17	644	34.33	860	33.48
**Total**	**693**	**100.00**	**1876**	**100.00**	**2569**	**100.00**

*MPMycoplasma* pneumoniae

The air pollutant measurements in Shanghai covered a span of 4 years (2015–2018) and encompassed 1461 daily measurements (shown in Table 2), as well as the relevant 4-year weather conditions. As shown in Table 2, during the study period of January 2015 through December 2018, the mean daily ambient concentrations of PM_2.5_, PM_10_, O_3_, SO_2_, and NO_2_ in Shanghai were 42.93 ± 28.95, 58.60 ± 32.65, 105.04 ± 45.93, 13.23 ± 6.89, and 43.62 ± 20.03 μg/m^3^, respectively, and that of CO was 0.77 ± 0.27 mg/m^3^.

**Table 2:** Distribution of daily ambient concentrations and daily temperatures in Shanghai, China, 2015–2018 (*n* = 1461)

Variable	X ± S	Min	P25	P50	P75	Max	IQR
PM_2.5_(μg/m^3^)	42.93 ± 28.95	5.00	22.00	35.00	55.00	218.00	33.00
PM_10_(μg/m^3^)	58.60 ± 32.65	8.00	36.00	50.00	74.00	250.00	38.00
O_3_(μg/m^3^)	105.04 ± 45.93	11.00	73.00	97.00	131.00	286.00	58.00
SO_2_(μg/m^3^)	13.23 ± 6.89	4.00	9.00	11.00	15.00	75.00	6.00
NO_2_(μg/m^3^)	43.62 ± 20.03	6.00	29.00	40.00	55.00	139.00	26.00
CO(mg/m^3^)	0.77 ± 0.27	0.40	0.60	0.70	0.90	2.20	0.30
T (°C)	18.43 ± 8.71	−5.00	11.00	19.50	25.50	35.50	14.50
H (%)	70.77 ± 6.31	53.00	66.00	71.00	75.00	83.00	9.00

X, mean value; S, standard deviation; Min, minimum value; Max, maximum value; IQR, interquartile range; PM_2.5_, fine particulate matter; PM_10_, particulate matter; O_3_, ozone; SO_2_, sulfur dioxide; NO_2_, nitrogen dioxide; CO, carbon monoxide; T, daily mean temperature; H, relative humidity

The daily measurements of ambient air pollutants during the 4-year period revealed that the PM_2.5_, PM_10_, O_3_, NO_2_, SO_2_, and CO concentrations surpassed the allowed limit in Shanghai on 725 days (49.62%), 717 days (49.08%), 675 days (46.20%), 83 days (5.68%), 8 days (0.55%), and 0 days (0.00%), respectively.

Pearson’s correlation coefficients among the air pollutants, daily mean temperature, and monthly relative humidity in Shanghai are displayed in Table 3. Significant correlations were found among the air pollutants in Shanghai, especially between PM_2.5_ and PM_10_ (r = 0.864), PM_2.5_ and CO (r = 0.863), PM_10_ and SO_2_ (r = 0.745), PM_2.5_ and SO_2_ (r = 0.742), NO_2_ and CO (r = 0.739), PM_10_ and CO (r = 0.726), SO_2_ and CO (r = 0.714), NO_2_ and PM_2.5_ (r = 0.698), NO_2_ and PM_10_ (r = 0.648), and NO_2_ and SO_2_ (r = 0.637). A conspicuous elevation in the daily air pollutant levels was observed with reductions in the daily mean temperature and monthly relative humidity.

**Table 3:** Pearson’s correlation coefficients among air pollutants in Shanghai, China, 2015–2018 (*n* = 1461)

Variable	PM_2.5_	PM_10_	O_3_	SO_2_	NO_2_	CO	T	H
PM_2.5_	1.000	0.864**	0.058*	0.742**	0.698**	0.863**	− 0.293**	0.014
PM_10_	–	1.000	0.136**	0.745**	0.648**	0.726**	− 0.248**	− 0.065*
O_3_	–	–	1.000	−0.081**	− 0.199**	− 0.131**	0.537**	− 0.015
SO_2_	–	–	–	1.000	0.637**	0.714**	−0.415**	0.044
NO_2_	–	–	–	–	1.000	0.739**	−0.434**	−0.068**
CO	–	–	–	–	–	1.000	−0.333**	0.111**
T	–	–	–	–	–	–	1.000	0.233**
H	–	–	–	–	–	–	–	1.000

**Significant at *p* < 0.01. *Significant at p < 0.05. PM_2.5_, fine particulate matter; PM_10_, particulate matter; O_3_, ozone; SO_2_, sulfur dioxide; NO_2_, nitrogen dioxide; CO, carbon monoxide; T, daily mean temperature; H, monthly relative humidity.

### Association between various air pollutants and *Mycoplasma* pneumonia in children

In the single-pollutant models, the most notable lag periods for NO_2_ were lag 0 and lag 1 (OR = 1.005, 95% CI = 1.000–1.010), and the most apparent lag time for PM_2.5_ was lag 1 (OR = 1.003, 95% CI = 1.000–1.006). The single-pollutant model showed a 0.5% (95% CI, 1.000–1.010) enhanced risk of pediatric hospital admissions for *Mycoplasma* pneumonia per 10-μg/m^3^ increase in the NO_2_ level on lag 0. The model showed an increase of 0.5% on lag 1 as well. The model also showed a 0.3% (95% CI, 1.000–1.006) enhanced risk of pediatric hospital admissions for *Mycoplasma* pneumonia per 10-μg/m^3^ increase in the PM_2.5_ concentration on lag 1 (Table 4). The association between PM_10_ and pediatric hospital admissions for *Mycoplasma* pneumonia was not significant in single-pollutant models (*p* > 0.5). O_3_, SO_2_, and CO exhibited paradoxical patterns in the single-pollutant models.

**Table 4:** Association between daily concentrations of atmospheric pollutants and pediatric hospital admissions for *Mycoplasma* pneumonia in Shanghai, China, 2015–2018: single-pollutant models (*n* = 2569)

				OR	95%CI			
Air pollutants	Lag0	Lag1	Lag2	Lag3	Lag4	Lag5	Lag6	Lag7
PM_2.5_	1.002 (0.998–1.005)	1.003 (1.000–1.006)	0.998 (0.995–1.002)	0.998 (0.994–1.001)	0.998 (0.995–1.002)	1.001 (0.997–1.004)	1.000 (0.996–1.003)	1.001 (0.998–1.005)
PM_10_	1.001 (0.998–1.004)	1.001 (0.998–1.004)	0.999 (0.996–1.002)	0.998 (0.995–1.001)	0.998 (0.995–1.001)	1.001 (0.998–1.003)	0.998 (0.995–1.002)	1.001 (0.998–1.004)
O_3_	0.997* (0.994–1.000)	0.996* (0.993–0.999)	0.998 (0.995–1.000)	1.000 (0.997–1.002)	1.000 (0.997–1.003)	1.002 (1.000–1.005)	0.998 (0.996–1.001)	0.999 (0.997–1.002)
SO_2_	0.982 (0.962–1.002)	0.986 (0.966–1.007)	0.978 (0.959–0.998)	0.968 (0.949–0.988)	0.967* (0.947–0.988)	0.984 (0.965–1.005)	0.978 (0.958–0.997)	0.978 (0.959–0.998)
NO_2_	1.005* (1.000–1.010)	1.005* (1.000–1.010)	1.002 (0.997–1.006)	1.000 (0.995–1.005)	1.000 (0.995–1.005)	1.001 (0.996–1.006)	1.000 (0.995–1.005)	1.001 (0.996–1.005)
CO	1.046 (0.721–1.517)	1.232 (0.850–1.786)	0.735 (0.501–1.076)	0.696 (0.481–1.008)	0.689* (0.480–0.990)	0.939 (0.651–1.355)	0.873 (0.592–1.289)	1.006 (0.685–1.477)

Adjusted for sex, age, season, daily mean temperature, and relative monthly humidity

*OR* odds ratio; *CI* confidence interval; *PM*_*2.5*_ fine particulate matter; *PM*_*10*_ particulate matter; *O*_*3*_ ozone; *SO*_*2*_ sulfur dioxide; *NO*_*2*_ nitrogen dioxide; *CO* carbon monoxide

Lag 0, day of hospital admission; Lag 1, day before hospital admission; and so forth

*Significant at *p* < 0.05

Multi-pollutant models were applied to ensure the stability of the latent effects of NO_2_ and PM_2.5_. Both NO_2_ and PM_2.5_ remained significantly associated with pediatric hospital admissions for *Mycoplasma* pneumonia after inclusion of the other pollutants (PM_10_, O_3_, SO_2_, and CO) into the models. The multi-pollutant models showed a positive association between NO_2_ and pediatric hospital admissions for *Mycoplasma* pneumonia on lag 0, lag 2, lag 3, and lag 4 after controlling for meteorological variables. A positive association was found between PM_2.5_ and pediatric hospital admissions for *Mycoplasma* pneumonia on lag 1 (Table 5). The association between PM_10_ and pediatric hospital admissions for *Mycoplasma* pneumonia was also not significant in multi-pollutant models (*p* > 0.5). The other pollutants in the multi-pollutant models were not risk factors in our analysis.

**Table 5:** Association between daily concentrations of atmospheric pollutants and pediatric hospital admissions for *Mycoplasma* pneumonia in Shanghai, China, 2015–2018: multi-pollutant models (*n* = 2569)

	NO_2_^a^		PM_2.5_^a^	
	OR	95%CI	OR	95%CI
Lag0	1.010*	1.003–1.018	1.006	0.998–1.014
Lag1	1.005	0.997–1.012	1.009*	1.000–1.017
Lag2	1.009*	1.001–1.016	1.003	0.994–1.011
Lag3	1.009*	1.002–1.017	1.002	0.994–1.011
Lag4	1.009*	1.002–1.016	1.006	0.997–1.015
Lag5	1.005	0.998–1.012	1.000	0.992–1.008
Lag6	1.003	0.995–1.010	1.006	0.997–1.015
Lag7	1.001	0.993–1.008	1.004	0.995–1.013

Adjusted for sex, age, season, daily mean temperature, and relative monthly humidity

*OR* odds ratio; *CI* confidence interval; *NO*_*2*_ nitrogen dioxide; *PM*_*2.5*_ fine particulate matter

^a^In the multi-pollutant model, which includes PM_2.5_, PM_10_, O_3_, SO_2_, NO_2_, and CO

*Significant at *p* < 0.05

## Discussion

This study is one of the few to investigate the association between exposure to air pollutants and pediatric hospital admissions for *Mycoplasma* pneumonia, especially in a typically populous metropolis (Shanghai, China). Our data revealed that the NO_2_ and PM_2.5_ concentrations were positively associated with the increases in daily pediatric hospital admissions for *Mycoplasma* pneumonia with hysteresis effects in both single-pollutant and multi-pollutant models.

Previous investigations showed variations in the associations between hospital admissions and the lag times of atmospheric pollutant levels (ranging from the day of admission to the previous 7 days). We found very few studies with longer lag times. Therefore, we investigated the air quality measures with a 7-day lag time. Most of the statistically significant positive associations were found between the 0-day to 4-day lagged air quality metrics (especially NO_2_ and PM_2.5_) and pediatric *Mycoplasma* pneumonia. No obvious associations were found between PM_10_, SO_2_, or CO and pediatric *Mycoplasma* pneumonia for any of the 7-day lags in this study. A pronounced association was detected between NO_2_ exposure and pediatric hospital admissions for *Mycoplasma* pneumonia, revealing NO_2_ exposure as a risk factor on lag 0 and lag 1 in the single-pollutant models and on lag 0, lag 2, lag 3, and lag 4 in the multi-pollutant models. In 2016, de Souza and Nascimento reported NO_2_ as a risk factor for pediatric hospitalization for pneumonia on lag 1 and lag 5, with a 10-μg/m^3^ increase in the concentration of this pollutant leading to a 7% elevation in the relative risk [19]. In 2018, Carvalho et al. examined more than 150 pediatric hospital admissions for respiratory diseases including pneumonia, bronchitis-bronchiolitis, asthma, and laryngitis-tracheitis. They found that NO_2_ was a significant risk factor on lag 2 and lag 3 in a single-pollutant model. They also found a positive association on lags 2 to 5 and lag 7 with a relative risk of 1.05 to 1.09 per 10-μg/m^3^ increase in the NO_2_ concentration [20]. A meta-analysis of 10 European birth cohorts within the ESCAPE project detected consistent evidence that the combined adjusted ORs for pneumonia were significantly higher for NO_2_ (OR = 1.30 and 95% CI = 1.02–1.65 per 10-μg/m^3^ increase in NO_2_) in early childhood [21]. In 2014, Lu et al. reported a significant association between an elevated risk of pneumonia in children and increased levels of NO_2_ (OR = 1.157, 95% CI = 1.121–1.195) as well as the episode day (OR = 1.038, 95% CI = 1.024–1.051) [22]. In accordance with our findings, the results of a study conducted in Jinan showed a positive association between the NO_2_ concentration and pediatric hospital admission for pneumonia, with a higher mean daily NO_2_ concentration (55.2 ± 22.4 μg/m^3^) than in our study [23]. Notably, the mean daily NO_2_ concentration in Shanghai (43.62 ± 20.03 μg/m^3^) was slightly lower than that in Changsha (46 μg/m^3^), while the study carried out in Changsha failed to discern an association between exposure to NO_2_ and pediatric hospital admissions for pneumonia [24].

The latent mechanisms by which ambient air pollution threatens the respiratory health of children have not been elucidated until now. As a free radical, NO_2_ can give rise to injuries and inflammation by means of depleting tissue antioxidant defenses. Numerous experimental studies have indicated that NO_2_ exerts a range of detrimental effects on lung metabolism, structure, function, inflammation, and host defenses against pulmonary infections. Berglund et al. reported that lung susceptibility to bacterial and viral infections is increased due to exposure to NO_2_, which may provide a breeding bed for *M. pneumoniae* [25]. Acute exposure to higher levels of NO_2_ can attenuate pulmonary bactericidal activity and alveolar macrophage function, suggesting that specific host defense functions can be obstructed due to NO_2_. Moreover, investigations have detected nitric and nitrous acids or their salts in the blood and urine, implying that NO_2_ or its chemical products can remain within the lung for prolonged periods. This might be a conceivable explanation for the observed lag effects of NO_2_ in the present study [26, 27]. As one of the main anthropogenic emission approaches, the combustion processes in mobile sources (internal combustion engines in vehicles and ships) account for one of the major sources of NO_2_ in Shanghai. Rush-hour traffic emissions of NO_2_ can reach relatively high levels even exceeding 940 μg/m^3^ [28]. Svartengren et al. reported that the NO_2_ concentration inside a car in a road tunnel during rush hour could reach 179 to 668 μg/m^3^ [29]. Living in such a traffic-clogged city in China, the population in Shanghai can inevitably suffer from the air pollution caused by automobile exhaust emissions. For children, who are particularly sensitive to the external environment, the risk of *Mycoplasma* pneumonia associated with air pollution is undoubtedly increased. This can be attributed to their immature immune system and lung function, as well as their high respiratory rates.

Our study also showed that the average PM_2.5_ concentration exceeded 35 μg/m^3^ (24-h limit in China), which may trigger notably deleterious health effects in children with *Mycoplasma* pneumonia. Few similar associations have been reported to date. In 2010, Belleudi et al. found that a 10-μg/m^3^ increase in the PM_2.5_ concentration was positively associated with a 3.04% (95% CI = 0.83–5.30) increase in hospital admissions for lower respiratory tract infections on lag 3 [30]. In 2016, Patto et al. reported that a 10-μg/m^3^ increase in the PM_2.5_ concentration led to a significant elevation of 25 to 28 ppb in the risk of hospitalization for pneumonia among children 4 and 5 days after exposure. The data in the present study showed a 0.9% increase in pediatric hospital admissions for *M. pneumoniae* infection per-10 μg/m^3^ increase in the PM_2.5_ concentration on lag 1 in the multi-pollutant models. In our study, it was found that Pearson’s correlation coefficient of PM_2.5_ and PM_10_ was high. However, the results of the single-pollutant models showed a 0.3% (95% CI, 1.000–1.006) enhanced risk of pediatric hospital admissions for *Mycoplasma* pneumonia per 10-μg/m^3^ increase in the PM_2.5_ concentration on lag 1, while PM_10_ did not appear to be associated with pediatric hospital admissions for *Mycoplasma* pneumonia. Although PM_10_ contains not only particles with an aerodynamic diameter of 0.1 to 2.5 μm but also coarse PM with an aerodynamic diameter of 2.5 to 10 μm, PM_10_ still had no association with pediatric hospital admissions for *Mycoplasma* pneumonia in multi-pollutant models, indicating that the core element affecting pediatric hospital admissions for *Mycoplasma* pneumonia is fine PM (PM_2.5_) rather than PM_10_.

Numerous studies have revealed that inhaled particulate matter has detrimental consequences not only for the lungs but also for other organs and tissues. Controlled-exposure studies involving humans and animals have implied that ambient particles might have direct influences on the respiratory tract. Adverse reactions such as the occurrence of inflammation, attenuation of pulmonary defense function, and deterioration of preexisting respiratory diseases may be attributed to the above-mentioned impacts [31]. The potential mechanisms of the adverse respiratory impacts of exposure to PM_2.5_ may be that inhaled PM_2.5_ increases the generation of antigen-specific immunoglobulins, changes the airway reactivity toward antigens, or influences the ability of the lungs to deal with bacteria, which may account for the increased susceptibility to microbial infection such as that caused by *M. pneumoniae* [32]. Responses to PM_2.5_ via inflammatory mediators and oxidative stress represent an intricate mixture and may be relevant to the different materials absorbed in particles, such as metals, organic carbon, sulfates, nitrates, and other biogenic components [33]. For instance, mechanisms of oxidative stress induction and modulation of the host immune system and inflammatory responses via Toll-like receptors and/or the nuclear factor-kappa B pathway are postulated to be the result of PM_2.5_-associated metal complex interaction with *M. pneumoniae* [34]. Some of the components of PM_2.5_ that are deposited in the lung can be dissolved within seconds or minutes, while some of the dissolution processes can last for hours or days. Insoluble components can remain in the lung for months or even years, which may explain the impact of the lag time of PM_2.5_ in this study.

To a large extent, the generation of PM_2.5_ in developed countries can be traceable to anthropogenic sources. Predominant sources are the combustion of fossil fuels, biomass burning, and resuspended soil dust. In contrast, predominant sources of ambient PM_2.5_ in developing countries include vehicle emission and biomass smoke. In Shanghai, fuel emissions from mobile sources and exhaust emissions from industrial manufacturing account for most local sources. Considering their more frequent physical activity and longer time spent outside, children may have more access to ambient PM_2.5_ than adults. Children are one of the most susceptible groups to the effects of air pollutants. Higher respiratory rates undoubtedly result in a higher intake of air pollutants. Moreover, children’s developing lungs may be less capable of handling toxic invasion because of their limited metabolic capacities. The increased susceptibility to *M. pneumoniae* can also be ascribed in part to their immature immune systems.

The analytic results of the single-pollutant models and multi-pollutant models demonstrated that the other air pollutants were not risk factors in our study. At the outset, the results of the single-pollutant models showed that O_3_, SO_2_, and CO seemed to be protective factors based on their association with pediatric hospital admissions for *Mycoplasma* pneumonia. However, after further analysis of all six air pollutants, O_3_, SO_2_, and CO were no longer potential protective factors. Consistent with our results of O_3_ in the single-pollutant models, O_3_ also displayed a paradoxical pattern in the analysis performed by de Souza and Nascimento [19]. The reason for this paradoxical pattern is unknown and requires further study.

The present study has several limitations. First, the Shanghai population data were obtained from only one institution, which might not be representative of the whole population of Shanghai. Second, exposure measurement errors resulting from discrepancies between the population-average exposure and atmospheric pollutant levels were unavoidable and might have incurred bias toward null and underestimated associations. Third, individuals’ exposure to air pollutants was not taken into account. The exposure levels were deemed homogenous throughout the city. Fourth, we did not thoroughly investigate the possible association between indoor air pollution and *Mycoplasma* pneumonia in children. The latent influence of such pollution, which may be caused by house decoration, tobacco smoke, and strong oil fumes from cooking, may be underestimated. Fifth, although the time lag effects during the *M. pneumoniae* invasion period were taken into consideration, the admission day for *Mycoplasma* pneumonia in children might not be the first day that symptoms occurred. They may have previously received emergency treatment. This might have introduced bias. Erroneous diagnosis and double counting of the same patient may have also occurred. Further in-depth studies are needed to clarify the mechanisms underlying the potential association between pediatric *Mycoplasma* pneumonia and ambient air pollutants.

## Conclusions

This study provided evidence of positive associations between elevated NO_2_ and PM_2.5_ concentrations and higher daily numbers of hospital admissions for *Mycoplasma* pneumonia in children in Shanghai. Of all parameters studied, NO_2_ and PM_2.5_ had statistically significant associations with hospital admissions and might be risk factors for Mycoplasma pneumonia in children. The strength of the associations between the NO_2_ and PM_2.5_ concentrations and pediatric admissions for *Mycoplasma* pneumonia might be influenced by the levels of the above-described pollutants as well as the hysteresis effect. These findings indicate that the high incidence of *Mycoplasma* pneumonia in children in Asia might be attributed to the heavy air pollution in Asia. The results of our study highlight the imperative need for public health policies, especially in highly polluted areas in Asia. More vigorous actions to reduce the air pollutant levels are also in urgent need to protect and promote public health, particularly for children.

## Data Availability

The datasets analyzed during the current study are available from the corresponding author on reasonable request and with permission of the related health department.

## Abbreviations

CI: Confidence interval
CO: Carbon monoxide
NO_2_: Nitrogen dioxide
O_3_: Ozone
OR: Odds ratio
PM_10_: Particulate matter (particles of < 10 μm in aerodynamic diameter)
PM_2.5_: Fine particulate matter (particles of < 2.5 μm in aerodynamic diameter)
ppb: Parts per billion
SO_2_: Sulfur dioxide
